# Interaction of anxiety and hypertension on quality of life among patients with gynecological cancer: a cross-sectional study

**DOI:** 10.1186/s12888-023-04521-5

**Published:** 2023-01-11

**Authors:** ZhiHui Gu, ChenXin Yang, Lin Tang, Hui Wu

**Affiliations:** Department of Social Medicine, School of Health Management, China Medical University, Shenyang North New District, No. 77 Puhe Road, Shenyang, Liaoning 110122 People’s Republic of China

**Keywords:** Gynecological cancer, Quality of life, Anxiety, Social support, Hypertension

## Abstract

**Background:**

Patients with gynecological cancer are prone to anxiety, and many of them are accompanied by hypertension, which seriously affects the quality of life (QOL). The study was to explore the interaction of anxiety and hypertension on QOL, and the moderating effect of perceived social support (PSS) in the impact of anxiety and hypertension on QOL of patients with gynecological cancer.

**Methods:**

A cross-sectional study was conducted in 2020, and 566 patients have been collected from the Affiliated Hospital of China Medical University. The Self-Rating Anxiety Scale (SAS), the Functional Assessment of Cancer Therapy Genera tool (FACT-G), and the Multidimensional Scale of Perceived Social Support Scale (MSPSS) were used. The interaction was analyzed by additive model, and the moderating effect was conducted by regression analysis and the simple slope analysis.

**Results:**

We found that 68.8% of patients had poor QOL due to the interaction between anxiety and hypertension. The relative excess risk ratio (RERI) was 22.238 (95%CI:44.119–88.596); the attribution ratio (AP) was 0.688 (95%CI:0.234–1.142); The interaction index (S) was 3.466 (95%CI: 0.823–14.435). The interaction items of PSS and anxiety were negatively correlated with QOL (β = -0.219, *P* < 0.01) and explained an additional 4.0% variance (F = 68.649, Adjusted R^2^ = 0.399, ΔR^2^ = 0.040, *P* < 0.01); PSS and blood pressure interaction item was not associated with QOL (β = 0.013, F = 55.138, Adjusted R^2^ = 0.365, ΔR^2^ = 0.001, *P* = 0.730).

**Conclusions:**

When anxiety and hypertension coexist, the QOL was affected. PSS played a moderating role in the impact of anxiety on QOL. Healthcare providers should take intervention measures to improve patients’ social support to reduce the impact of anxiety on QOL.

## Background

In recent years, the incidence rate of gynecological tumors including cervical cancer, endometrial cancer, ovarian cancer and other malignant tumors has been rising year by year, and ranked the third in female mortality in China[1]. Surgery combined with chemotherapy is an effective treatment for gynecological tumors. Although its prognosis and survival rate have been significantly improved, it inevitably brings physical pain and psychological changes to patients. The quality of life (QOL) is often better than the survival rate in reflecting the treatment outcome of cancer patients [2]. QOL is defined as the “individual’s perception of their position in life in the context of culture and value systems in which they live and in relation to their goals, expectations, standards and concerns” [3]. QOL evaluation is an important outcome indicator of cancer research, reflecting the changes of physiological, social, psychological and emotional aspects of patients after illness [4]. The course of disease, the loss of female characteristics after surgery and the accompanying symptoms of patients with gynecological cancer, such as sexual health, fertility and sexual desire problems, seriously affect the QOL of patients [5]. In addition to surgical treatment for cancer and other physiological factors, the negative psychological factors also have a negative impact on the QOL. Cancer patients not only suffer from physical pain, but also face complex psycho-social problems, which have a negative impact on the QOL [6].

### Influence of anxiety and hypertension on QOL

Among the psychological factors affecting the QOL, cancer-related anxiety is more common in the incidence of emotional disorders [7]. Sudden onset of disease or partial organ loss caused by tumor resection will affect the patients’ self-esteem and self-confidence, hence affect the patients’ self recognition, and cause severe anxiety. Previous studies have shown that the anxiety with female patients diagnosed with cancer in the preoperative, postoperative and chemotherapy period is widespread and the incidence is high [8]. The level of anxiety in female cancer patients was significantly higher than that in male cancer patients, which indicated that patients with gynecological cancer may belong to an anxious group [9, 10]. Studies have shown that the incidence of anxiety in patients with gynecological cancer is 23.7% to 42% [11–13]. Cancer patients have obvious psychological stress reaction or psychological disorder, especially anxiety, which will affect coping style, treatment compliance, immune function and reduce QOL [14]. A meta-analysis showed that anxiety affect 10% of cancer patients at any stage of cancer [15]. About 75% of patients with obvious anxiety did not receive any psychological or drug-related treatment systematically or never [16], leading to the obstruction of making anticancer decision, poor treatment compliance, prolonged disease recovery time, and negatively affected their QOL [17]. In cancer patients, the most common cardiovascular disease is hypertension. Previous study showed that compared with the normal population, the incidence rate of anxiety in patients who knew their blood pressure values were high [18]. Patients with hypertension may be excessively nervous due to their lack of understanding of the disease and worry about the adverse effects of anti hypertensive drug treatment, resulting in poor mood and anxiety. This psychological state in turn will aggravate the condition of hypertension, weaken the medication adherence, and cause a vicious circle between hypertension and anxiety [19]. The epidemiological investigation on hypertension and anxiety comorbidity showed that the prevalence of anxiety complicated with hypertension in China is 11.6%—38.5% [20]. A survey in Ghana found that 56.0% of hypertensive patients had anxiety [19]. Hypertension can affect the QOL of the elderly population, and has a greater impact on elderly women [21]. However, few studies have discussed the interaction effect of hypertension and anxiety on the QOL.

### The moderating effect of social support

For patients with anxiety and hypertension coexisting, it is inevitable that their QOL will be affected. In order to balance the impact of stressful life events, some studies have emphasized the importance of social support on the QOL of patients with mental illness [22]. Social support refers to the spiritual or material help and support system given by the outside world, and a good social support system helps to promote mental health [23]. Huang et al. (2013) found that social support was a moderator of depression on QOL in breast cancer patients, which can significantly alleviate the impact of depression on QOL [24]. Panayiotou et al. (2013) found that social support helps the negative impact of anxiety on QOL [25]. Anyway social support directly and indirectly regulates the influence of variables to play its role, that is, the “buffer hypothesis”, which has been widely confirmed [26]. Social support is a regulatory factor between stress and health, and helps individuals cope with crisis and better adapt to changes in the environment, and the effect of social support on health can only occur during the transition period of life trajectory and sudden crisis[27]. Previous research found that social support itself had no direct impact on health, but indirectly benefited physical health by buffering social pressure and psychological pressure, while the lack of social support would aggravate the negative impact of pressure on the body [28]. To summarize, social support can improve the individual’s sense of security and self-confidence in the face of stress by providing corresponding social resources or psychological resources for individuals under stress, so as to strengthen the stress ability of individuals, reduce the erosion of stress on individuals, and thus improve individual health. Therefore, this paper chose social support as the moderating variable in gynecologic tumor patients with hypertension and anxiety.

The purposes of this study are as follows: 1) This study analyzed the effect of the interaction of anxiety and hypertension on the QOL. 2) For patients with anxiety and hypertension, it also aims to test whether the social support could moderate the impact of anxiety and hypertension on QOL, and to provide the theoretical basis for improving the QOL of patients with gynecological cancer.

## Methods

### Study design and sample

Since December 2019 to July 2020, a total of 566 patients with gynecological cancer have been collected from the Affiliated Hospital of China Medical University in Liaoning, Shenyang. Inclusion criteria: during the investigation, the condition was relatively stable, with clear consciousness and no serious complications; voluntary participants; the expected survival time was > 9 months.

### Measurement of general characteristics of patients

General characteristics included age, marital status, education level, monthly income (CNY), habitation, hypertension, stage of cancer, BMI. The measurement of monthly income was based on the amount of fixed monthly income filled by the patients (1 CNY≈ 0.1397 USD). The hypertension was measured by the hospital nurses. Participants were asked to sit for at least 10 min. Supine brachial SBP and DBP were measured twice in the right arm for each subject. The mean score of the last two measurements was considered as the BP value. If the blood pressure was ≥ 140/90 mmHg, the subject was asked to rest for 15 min, and BP was retaken twice. The mean of the two measurements was the final result. SBP ≥ 140 mmHg and/or a DBP ≥ 90 mmHg was defined as Hypertension.

### Assessment of anxiety symptoms

Zung’s Self-Rating Anxiety Scale (SAS) was used [29]. The SAS considered both emotional and physical symptoms, including 20 items, of which 15 were negative experiences and 5 were positive experiences. The SAS uses a 4-point rating scale (1 = No or little; 2 = A little; 3 = Quite a bit; and 4 = Very much). Add all the items together to form a rough score, which is multiplied by 1.25 and rounded to get the standard score to evaluate anxiety. The index score of 45 (original score = 36) was set as the cut-off point for clinical significant anxiety in our study [30]. The Cronbach’s alpha of it was 0.918 which proved that the scale had good reliability.

### Assessment of quality of life

The Functional Assessment of Cancer Therapy Genera (FACT-G) tool is applicable to all cancer sites, and is included as the common cancer core questionnaire for specific instruments of each fact cancer site [31]. The FACT-G is a cancer-targeted QOL measure that includes physical well-being, social well-being, emotional well-being, and functional well-being. The FACT-G uses a 5-point rating scale (0 = Not at all; 1 = A little bit; 2 = Somewhat; 3 = Quite a bit; and 4 = Very much). The scale consists of 27 items with a total score range of 0 to 108. In this study, the Cronbach’s alpha of global scale was 0.899 which proved that the scale had good reliability.

### Assessment of perceived social support

The Multidimensional Scale of Perceived Social Support (MSPSS) is a subjective assessment of social support adequacy with 12-item with a total score range of 12–84[32]. The 12 items in MSPSS are divided into three sources of support, including family support, friend support and other support. The MSPSS uses a 7-point rating scale (1 = Strongly disagree; 2 = Disagree; 3 = Disagree slightly; 4 = Neutral; 5 = Agree slightly; 6 = Agree; 7 = Strongly agree). Add all the items together to form a rough score. In this study, the Cronbach’s alpha of global scale was 0.963 which proved that the scale had good reliability.

### Data analysis

SPSS Statistics 21.0 software was used for statistical analysis. Chi square test was used to compare the count data. The significant variables in univariate analysis were used as independent variables, and multiple logistic regression was performed. By using Delta method, the excel table compiled by Andersson et al. [33] was introduced to calculate the related indexes of additive interaction. The OR value obtained by logistic regression model in the interaction calculation process was used as the estimation value of relative risk (RR). Additive interaction index: (1) the relative excess risk due to interact (RERI) was used to evaluate the difference between the combined effect of factor A (anxiety) and factor B (hypertension) and the sum of factors A (anxiety) and B (hypertension) alone; (2) the attributable proportion due to interaction (AP) was used to evaluate the proportion of interaction between two factors when two factors A (anxiety) and B (hypertension) exist at the same time; (3) the synergy index (S): the confidence interval of S is greater than 1 [34–36]. Correlations among variables were examined by Pearson’s correlation. Hierarchical regression analysis was used to prove the relationship of variables and to examine the moderating effect. Finally, the simple slope analysis was conducted to visualize the interaction term [37]. Significance level was α = 0.05, and a two-tailed *P* < 0.05 was considered to have statistical significance.

## Results

### Basic information

566 cases were investigated with an average age of (56.34 ± 9.78) years. The average score of QOL was 72.69 ± 18.10. There were 304 cases with good QOL(> 72.69) and 262 cases with poor QOL(≤ 72.69). 253 cases had anxiety (44.7%); 192 cases had hypertension (33.9%); 113 cases had anxiety with hypertension (20.0%). The univariate analysis results between QOL and all categorical variables were shown in Table 1. Monthly income (CNY), hypertension, BMI, anxiety symptoms and perceived social support (PSS) were significantly correlated with QOL (*P* < 0.05). Age (Years), marital status, education level, habitation, and stage of cancer were not significantly correlated with QOL (*P* > 0.05).

**Table 1:** Comparison of basic conditions of patients with different QOL (*N* = 566)

Variables	Good	Poor	*χ*^*2*^	*P*
Age (Years)			3.528	0.171
< 50	55	60		
50–60	141	126		
≥ 60	108	76		
Marital status			0.681	0.409
Married/cohabited	33	23		
Single /separated	271	239		
Education level			4.722	0.094
Junior high school and below	170	170		
High/Technical secondary school	116	80		
Bachelor degree or above	18	12		
Monthly income (CNY)			6.112	0.047
< 2000	78	62		
2000–4000	163	164		
> 4000	63	36		
Habitation			3.636	0.057
Rural	199	191		
Urban	105	71		
Hypertension			56.254	< 0.001
No	243	131		
Yes	61	131		
Stage of cancer			5.034	0.081
I	163	122		
II	61	73		
III+ IV	80	67		
BMI			11.391	0.001
< 24	178	189		
≥ 24	126	73		
Anxiety			144.458	< 0.001
No	239	74		
Yes	65	188		
PSS			61.869	< 0.001
Low/Moderate	49	122		
High	255	140		

“Good” includes “fairly good” and “very good”, “Poor” includes “fairly bad” and “very bad” responses. Anxiety symptoms: No means the index score < 45, Yes means the the index score ≥ 45

*Abbreviation: BMI* Body mass index, *PSS* Perceived social support

### Multivariate logistic regression analysis of the factors affecting the QOL

Taking the QOL as the dependent variable, the variables with *P* < 0.1 in the single factor were included in the multiple logistic regression. The results showed that the factors that entered the equation were monthly income (CNY), hypertension, anxiety, and PSS. Monthly income > 4000 yuan and PSS were the protective factors for the QOL, and hypertension and anxiety were the risk factors for the QOL. The details were shown in Table 2.

**Table 2:** Multiple logistic regression analysis of QOL

Factors	B	S.E	Wald χ2	*P*	OR	95%CI
Monthly income (CNY)						
2000–4000 v s < 2000	-0.068	0.283	0.058	0.810	0.934	0.537–1.625
> 4000 vs 2000	-0.890	0.367	5.889	0.015	0.411	0.200–0.843
BMI	-0.078	0.223	0.123	0.726	0.925	0.597–1.433
Hypertension	1.229	0.221	30.888	< 0.001	3.419	2.216–5.273
Anxiety	1.945	0.230	71.584	< 0.001	6.994	4.457–10.975
PSS	-0.692	0.242	8.189	0.004	0.501	0.312–0.804
Constant	-0.832	0.389	4.575	0.032	0.435	

*Abbreviation: BMI* Body mass index, *PSS* Perceived social support

### Calculation of additive interaction index of anxiety and hypertension on QOL

As shown in Table 3, 113 patients (20.0%) were anxiety with hypertension. The reference were no anxiety and no hypertension. The OR value of hypertension without anxiety on QOL was 3.112 (95%CI:1.778–5.447); The OR value of anxiety without hypertension on QOL was 7.978 (95%CI:4.939–12.886); The OR value of hypertension and anxiety on QOL was 32.327 (95%CI: 16.848–62.026). The interaction between anxiety and hypertension was significant. The relative excess risk ratio (RERI) was 22.238 (95%CI:44.119–88.596); the attribution ratio (AP) was 0.688 (95%CI:0.234–1.142); The interaction index (S) was 3.466 (95%CI: 0.823–14.435). AP was 0.688, indicating that 68.8% of the patients with poor QOL due to the interaction between anxiety and hypertension.

**Table 3:** Analysis of interaction between anxiety symptoms and hypertension on QOL

Anxiety	Hypertension	QOL		*β*	*P*	OR/RR	95%CI
		Good	Poor				
-	-	192	42	-			
-	+	47	32	1.135	< 0.001	3.112	1.778–5.447
+	-	51	89	2.077	< 0.001	7.978	4.939–12.886
+	+	14	99	3.476	< 0.001	32.327	16.848–62.026
RERI						22.238	44.119–88.596
AP						0.688	0.234–1.142
S						3.446	0.823–14.435

“Good” includes “fairly good” and “very good”, “Poor” includes “fairly bad” and “very bad” responses

*Abbreviation: RERI* the relative excess risk ratio, *AP* the attribution ratio, *S* the interaction index

### Correlations among continuous variables

As shown in Table 4, anxiety was negatively correlated with QOL and PSS, and positively correlated with BP (*P* < 0.01). BP was negatively correlated with PSS and QOL (*P* < 0.01). PSS was positively correlated with QOL (*P* < 0.01).

**Table 4:** Correlations among study variables

Variables	Mean ± SD	1	2	3	4
Anxiety	47.43 ± 14.45				
BP	124.94 ± 23.25	0.140^**^			
PSS	68.11 ± 12.12	-0.539^**^	-0.246^**^		
QOL	72.69 ± 18.10	-0.473^**^	-0.349^**^	0.560^**^	1

*Abbreviation: BP* Blood pressure, *PSS* Perceived social support

^**^P < 0.01; 0.1 <|r|< 0.3 represents small/weak correlation, 0.3 <|r|< 0.5 represents medium/moderate correlation, 0.5 <|r| represents large/strong correlation

### Hierarchical regression analysis

As shown in Table 5, age, monthly income (CNY), and BMI were added in the first step. In the second block, anxiety and PSS were added. Finally, the PSS&Anxiety interaction term were added in the last block. The PSS&Anxiety interaction term was negatively correlated with QOL (*β* = -0.219, *P* < 0.01), and explained an extra 4.0% of the variance (F = 68.649, Adjusted R^2^ = 0.399, ΔR^2^ = 0.040, *P* < 0.01); The PSS&BP interaction term was not associated with QOL (β = 0.013, F = 55.138, Adjusted R^2^ = 0.365, ΔR^2^ = 0.001, *P* = 0.730).

**Table 5:** Hierarchical linear regression for anxiety symptoms and PSS with quality of life

Variables	Quality of Life		
	Block 1	Block 2	Block 3
Age	0.052	0.021	0.004
Monthly income (CNY)	0.057	0.101^**^	0.082^*^
BMI	0.110^**^	-0.026	-0.047
Anxiety		-0.256^**^	-0.220^**^
PSS		0.430^**^	0.373^**^
PSS&Anxiety			-0.219^**^
F	3.546^*^	64.673^**^	63.459^**^
Adjusted R^2^	0.013	0.360	0.399
ΔR^2^	0.019	0.347	0.040

*Abbreviation: BMI* Body mass index, *PSS* Perceived social support

^*^P < 0.05, **P < 0.01

### Simple slope analysis

**Fig. 1:**
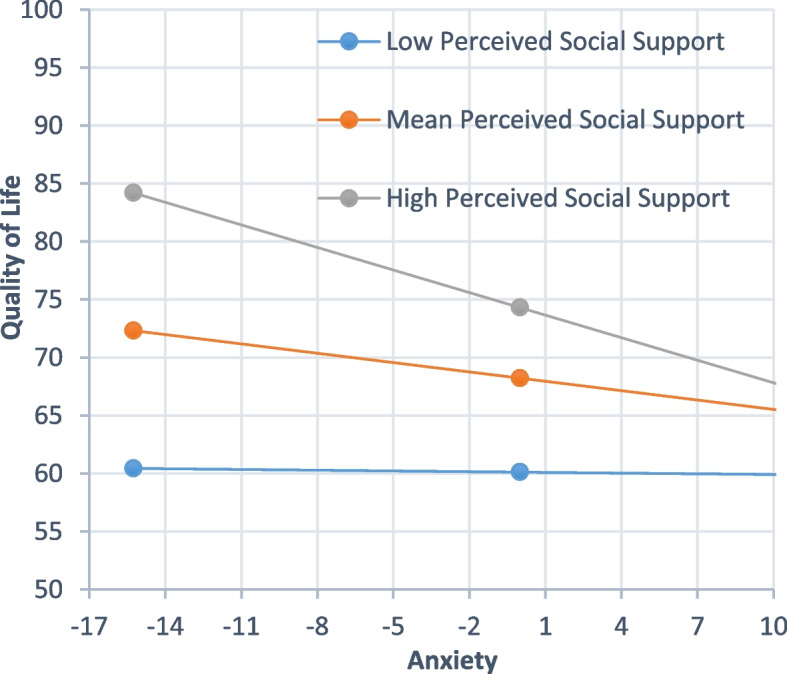
Simple slope plot of interaction between anxiety symptoms and PSS on QOL. Notes: Low, -1SD below the mean; High, + 1SD above the mean

In Fig. 1, simple slope analysis showed that the association between anxiety and QOL was gradually decreased in the low (-1SD below the mean, B = 0.110, β = 0.093, *P* < 0.05), mean (B = -0.269, β = -0.229, *P* < 0.01) and high (+ 1SD above the mean, B = -0.648, β = -0.552, *P* < 0.01) groups of PSS.

## Discussion

The results of this study showed that the interaction between anxiety and hypertension had a serious impact on the QOL of patients with gynecological cancer, and the impact of two factors was significantly greater than that of a single factor. Anxiety made patients with gynecological cancer have a low evaluation of their overall health, often showing physical symptoms, and their social function was significantly reduced. A study on patients with chronic hepatitis showed that mental components of health related QOL were predominantly effected by psychiatric conditions [38]. Therefore, the QOL of patients with anxiety symptoms was poor. Hypertension can significantly affect the QOL of elderly women, which has been concluded in previous studies [21]. Therefore, the QOL of patients with hypertension was poor. Hypertension is an important cardiovascular risk factor in women [39]. Especially in cancer patients, those with pre-existing hypertension have a poorer outcome, which might affect the QOL[40]. In this study, 68.8% of patients had poor QOL due to the interaction between anxiety and hypertension. There is an interactive relationship between hypertension and anxiety [41]. Longitudinal data and theoretical literature indicate that anxiety may precede hypertension [42]. Patients with anxiety are in low mood or pessimism for a long time due to unstable mood, which will lead to unstable blood pressure and high blood pressure. Patients with anxiety had higher rates of uncontrolled blood pressure, and had an increased risk of cardiovascular events. [43]. Researches also showed that the change process of hypertension will participate in and affect the generation of anxiety, and the fluctuation range of blood pressure and heart rate variability are positively related to the severity of anxiety [18, 44]. Therefore, hypertension will lead to anxiety, and anxiety will aggravate the condition of hypertension, leading to a vicious circle between hypertension and anxiety, which will affect the prognosis of the disease, cause serious physical and mental consequences. This was also the reason why the patient’s QOL was seriously reduced when the two coexist.

The results of this study showed that perceived social support had a moderating role in the impact of anxiety on the QOL of patients with gynecological cancer, and perceived social support can alleviate the impact of anxiety on the QOL of patients. Research has confirmed that social support can be used as personal and internal resources to cope with and adapt to stress situations, enabling people to explain and deal with diseases, difficulties, hopes and rehabilitation [45]. Social support is closely related to the physiological and psychological aspects of long-term survival of patients with gynecological cancer [46]. At the same time, social support is an important factor in predicting the QOL of cancer patients [47]. Research also shows that only when social support is needed, can social support relieve the anxiety of cancer patients [48]. Social support related research found that emotion is almost the common element of all social support. When people talk about social support, it often contains the emotional dimension. Only when the actual social support is perceived can the individual’s psychological adaptability be improved, therefore, the actual social support can be truly effective only when it is perceived emotionally[49]. This can explain why the more social support patients receive during treatment, the more beneficial it is to reduce their anxiety [50]. And it also explained why in our study, the relationship between anxiety and QOL was not obvious with lower perceived social support, but it is only with higher perceived social support that it played a moderating role. Based on the above theory, when patients with gynecological cancer have anxiety, higher social support can improve their impact on QOL by reducing the possibility of anxiety. The results of this study show that social support has no regulatory role in the impact of hypertension on the QOL of patients with gynecological cancer, which may be related to the pathogenesis of hypertension, and the use of hypertension drugs may achieve better control effects.

### Strengths and limitations

Our study included more participants than previous studies; Different from previous single factor studies, we analyzed the interaction between hypertension and anxiety on quality of life; Finally, we also analyzed the moderating effect of perceived social support to better explain the relationship between variables. This study was a cross-sectional study, so we could not get the causal relationship between variables; More variables that may affect the quality of life should be included; Self filled questionnaire will bring bias problems to this study, such as recall bias, measurement bias, etc.

## Conclusions

Our study found that anxiety and hypertension had a negative impact on the QOL, and the interaction of anxiety and hypertension had a greater impact on the QOL of patients with gynecological cancer than that of single factor. At the same time, perceived social support alleviated the impact of anxiety on the QOL. It is suggested that healthcare providers should pay more attention to patients with anxiety and hypertension, and take intervention measures to improve the perceived social support of patients with gynecological cancer, so as to improve the QOL.

## Data Availability

The datasets generated and/or analysed during the current study are not publicly available due the data also forms part of an ongoing study but are available from the corresponding author on reasonable request.
